# Assessment of mental wellbeing of undergraduate pharmacy students from 14 countries: The role of gender, lifestyle, health-related, and academic-related factors

**DOI:** 10.3389/fpubh.2022.1011376

**Published:** 2022-11-01

**Authors:** Mohamed Hassan Elnaem, Naeem Mubarak, Mohammed Salim K. T., Muna Barakat, Doaa H. Abdelaziz, Noha O. Mansour, Abrar K. Thabit, Diana Laila Ramatillah, Ali Azeez Al-Jumaili, Nabeel Kashan Syed, Mohammed Fathelrahman Adam, Md. Sanower Hossain, Mohamed A. Baraka, Jimmy Jose, Ramadan Elkalmi, Sarath Chandran, Inderpal Singh Dehele, Mahmoud Elrggal, Ahmed Ibrahim Fathelrahman

**Affiliations:** ^1^Department of Pharmacy Practice, Faculty of Pharmacy, International Islamic University Malaysia, Kuantan, Pahang, Malaysia; ^2^Department of Pharmacy Practice, Lahore Medical and Dental College, University of Health Sciences, Lahore, Pakistan; ^3^Department of Pharmacy Practice, Manipal College of Pharmaceutical Sciences, Manipal Academy of Higher Education, Manipal, Karnataka, India; ^4^Department of Clinical Pharmacy and Therapeutics, Faculty of Pharmacy, Applied Science Private University, Amman, Jordan; ^5^Pharmacy Practice and Clinical Pharmacy Department, Faculty of Pharmacy, Future University in Egypt, Cairo, Egypt; ^6^Department of Clinical Pharmacy, The National Hepatology and Tropical Medicine Research Institute, Cairo, Egypt; ^7^Clinical Pharmacy and Pharmacy Practice Department, Faculty of Pharmacy, Mansoura University, Mansoura, Egypt; ^8^Pharmacy Practice Department, Faculty of Pharmacy, King Abdulaziz University, Jeddah, Saudi Arabia; ^9^Faculty of Pharmacy, Universitas 17 Agustus 1945 Jakarta, Jakarta, Indonesia; ^10^College of Pharmacy, University of Baghdad, Baghdad, Iraq; ^11^Pharmacy Practice Research Unit, Department of Pharmacy Practice, College of Pharmacy, Jazan University, Jazan, Saudi Arabia; ^12^Faculty of Pharmacy, University of Science and Technology, Omdurman, Sudan; ^13^Centre for Sustainability of Ecosystem and Earth Resources (Pusat ALAM), Universiti Malaysia Pahang, Kuantan, Pahang, Malaysia; ^14^Clinical Pharmacy Program, College of Pharmacy, Al Ain University, Al Ain, United Arab Emirates; ^15^Clinical Pharmacy Department, College of Pharmacy, Al-Azhar University, Cairo, Egypt; ^16^Department of Pharmacy, University of Nizwa, Nizwa, Oman; ^17^Department of Pharmacology, Faculty of Medicine, Sebha University, Sebha, Libya; ^18^College of Pharmaceutical Sciences, Government Medical College Kannur, Kannur, Kerala, India; ^19^School of Pharmacy, University of Birmingham, Birmingham, United Kingdom; ^20^College of Pharmacy, Umm Al-Qura University, Makkah, Saudi Arabia; ^21^Department of Clinical Pharmacy, College of Pharmacy, Taif University, Taif, Saudi Arabia

**Keywords:** mental wellbeing, student, COVID-19, pandemic, pharmacy, education

## Abstract

**Background:**

Pharmacy students will assume future roles as frontline healthcare providers. Therefore, evaluating their current state of mental wellbeing and its associated factors is essential for better planning students' support initiatives. This study aimed to assess mental wellbeing and its associated factors among undergraduate pharmacy students from 14 countries during the pandemic.

**Methods:**

A cross-sectional study was conducted among undergraduate pharmacy students in 14 countries in Asia and the Middle East. The validated Warwick-Edinburgh Mental Wellbeing Scale (the 14-item WEMWBS) was adopted to assess mental wellbeing. Data collection was performed online between February and April 2022. Descriptive and inferential statistics were used as appropriate.

**Results:**

A total of 2,665 responses were received, mainly from females (68.7%) with a higher presence of private universities (59.1%). About 34.9% had low mental wellbeing levels, while 57 and 8.1% had medium, and high levels, respectively. Binary logistic regression showed that males (AOR: 1.34; CI 95%: 1.11–1.61; *p* < 0.01) and students with no chronic illnesses (AOR: 2.01; CI 95%: 1.45–2.80; *p* < 0.001) were more likely to have higher mental wellbeing. Also, participants who did not engage in any exercise (AOR: 0.71; CI 95%: 0.52–0.98; *p* = 0.04) and those in public universities (AOR: 0.82; CI 95%: 0.69–0.97; *p* = 0.02) were less likely to have higher mental wellbeing. Additionally, students who had interest/passion for pharmacy (AOR: 1.69; CI 95%: 1.07–2.68; *p* = 0.02), and those who known pharmacists inspired (AOR: 1.81; CI 95%: 1.06–3.12; *p* = 0.03), were more likely to have higher mental wellbeing compared with those who had no specific reason for their choice to study pharmacy. The participants with excellent (AOR: 1.87; CI 95%: 1.29–2.70; *p* = 0.001) or very good self-reported academic performance (AOR: 1.57; CI 95%: 1.12–2.22; *p* = 0.01) were more likely to have higher mental wellbeing compared to those with fair academic performance.

**Conclusion:**

More than a third of the participants had low mental wellbeing. Various demographic, lifestyle, medical and academic factors appeared to affect students' mental wellbeing. Careful consideration of these factors and their integration into the pharmacy schools' plans for student support services and academic advising would be essential to improve students' mental wellbeing.

## Introduction

Good mental health and wellbeing are crucial in achieving a better quality of life. There has been growing interest in measuring mental wellbeing, recognizing that mental health is more than the absence of mental illness (1). Mental wellbeing refers broadly to the individual capacity to maintain a state of feeling good and functioning well that is more than the outcome of treating or preventing mental illness (2). There has been a link between higher levels of mental wellbeing and positive health outcomes, such as lower risks for mental and physical disorders (3). The Warwick-Edinburgh Mental WellBeing Scale (WEMWBS) aims to broadly capture wellbeing, including affective-emotional aspects, cognitive-evaluative dimensions and psychological functioning (4). The scale is concise and positively rated all items to support mental health promotion initiatives (4). Focusing on university students' wellbeing was triggered by previous research that underpinned their stress experiences that continued throughout their academic life and be consistently higher than the stress experienced before university life (5). In addition, previous research highlighted that university students with low mental wellbeing were more prone to mental distress (6). Furthermore, maintaining higher psychological wellbeing was associated with decreasing the risk of stress experiences (7). Even before the pandemic, these observations warranted continuous efforts to improve university students' wellbeing.

During the pandemic, the global population, including students, has been adversely affected mentally due to several reasons, such as the associated uncertainty of the future, altered routines, financial losses, and social isolation that have escalated varieties of psychological issues involving depression, anxiety, and emotional breakdowns (8). Additionally, academic institutions were forced to close temporarily, and education was transformed through virtual or online mediums (9). The relatively new and less interactive learning mode appears to have created an additional challenge for most students who were adapted to the traditional common learning mode when virtual learning was introduced (10). Consequently, research has highlighted the negative impact on mental health among college students during this challenging time, demonstrated as facing academic difficulties and increased levels of mental distress (11). This negative impact could be further amplified if coupled with living and socioeconomic challenges (12).

In particular, assessing mental wellbeing among pharmacy students holds significant importance as they are a future pool of health care professionals. Recent data from a multicentre study among pharmacy students in the U.K. showed that their mental wellbeing was lower than that of other student populations (13). Also, previous evidence showed that pharmacy students, in particular, were at higher risk of anxiety and stigma regarding mental health treatment than their medical counterparts (14). Moreover, relatively recent evidence from New Zealand highlighted the impact of academic stress on students' mental wellbeing (15). However, there is a relative lack of comprehensive assessment of factors that may affect mental wellbeing among large and diverse pharmacy students in Asia and the Middle East. An extensive evaluation of mental wellbeing and its associated factors could help in better planning support initiatives for pharmacy students' academic health, which may impact their contribution to community health. Therefore, this study aimed to assess mental wellbeing and its associated factors among undergraduate pharmacy students in 14 countries during the COVID-19 pandemic.

## Methods

### Study design

This descriptive online cross-sectional study was conducted among pharmacy students in 14 countries from Asia and the Middle East: Bahrain, Bangladesh, Egypt, India, Indonesia, Iraq, Jordan, Libya, Malaysia, Oman, Pakistan, Saudi Arabia, Sudan, and the United Arab Emirates. The study used a validated, self-administered survey prepared in English and Arabic versions on Google Forms. It was disseminated through study co-investigators in each country via social media platforms to minimize face-to-face interactions and facilitate the process. Participants were asked to answer only one of the versions as per their convenience to avoid duplicate responses. The data was collected between February 1st and April 15th, 2022.

### Inclusion and exclusion criteria

Undergraduate pharmacy students who have studied for at least one semester in the pharmacy program in one of the participating countries were eligible to participate in the study. These eligible participants were registered and received their didactic learning content through face-to-face, virtual or hybrid modes. Internship (i.e., advanced pharmacy practice) students in Doctor of Pharmacy (PharmD) programs were excluded as they are not entirely under the didactic learning component in the pharmacy schools, so some of the questionnaire questions would not have fully applied to them.

### Sample size

This study was not directed to cross-country comparisons as there were considerable variations among countries regarding the number of pharmacy schools, target student population, and accessible sampling frames. Using the Raosoft sample size calculator, assuming an estimated proportion of 50% and a 95% confidence interval and confirming that at least one principal pharmacy school will be participating in each country, the minimum required sample size was estimated to be 377 students. As per the guidelines of sample size requirements for logistic regression analysis for observational studies, a minimum sample of 500 is required to drive statistics representing parameters in real-life data when eight independent variables are included (16). Therefore, the data collection was continued over 10 weeks to maximize participation from all countries and satisfy sample size requirements.

### Instrument structure, validity, and pilot testing

A 37-item questionnaire was used in this study. The questionnaire comprised three main parts. Part 1 included 11 items to cover the general socio-demographic details of the participants, such as age, gender, marital status, area of residence, monthly household income, and presence of chronic diseases. Then, Part 2 consisted of 12 statements to gather information on COVID-19 and learning experiences in the pandemic era, such as vaccination status, infection history, study year, academic performance, and reasons for choosing pharmacy program. Regarding academic performance, we provided definitions of each category in percentage corresponding to GPA on two scales of 4 and 5 to accommodate the differences between different schools in various countries. Finally, Part 3 used the fourteen-item validated Warwick-Edinburgh Mental Wellbeing Scale (14-item WEMWBS) to assess mental wellbeing (2).

We used the validated 14-item WEMWBS to assess mental wellbeing as described by the developer (2), which does not require further validation. WEMWBS is a 14-item scale covering subjective wellbeing and psychological functioning to address aspects of positive mental health (2). WEMWBS is a short, acceptable and meaningful tool to measure mental wellbeing that shows reliability, strong psychometric performance and lack of ceiling effects (4). All fourteen items are positively worded from 1 (none of the time) to 5 (all of the time). So, the total mental wellbeing scores were calculated from a minimum of 14 to a maximum of 70 for each participant. In addition, categorization of the total mental wellbeing scores was performed into three main categories, low (14–42) medium (43–60), and high (61–70) levels (1). WEMWBS was initially validated for use in the UK with those aged 16 and above, involving surveys in both student and general population samples (2). Currently, it has been widely used in diverse populations other than the UK and languages other than English (17–19).

However, for the remaining items in parts 1 and 2, we involved a panel of five experts in pharmacy practice in evaluating the questionnaire's content validity by estimating the content validity index for each item (I-CVI) to ascertain its relevance and clarity. The I-CVI should be at least 0.78 with a minimum of three experts (20). Any item with I-CVI < 0.78 for relevance was discarded from the questionnaire, while any item with I-CVI < 0.78 for clarity was improved for better clarity based on the experts' suggestions. The translation to the Arabic version was validated by forward-backward translation, starting with an English version that has been translated into Arabic, and the later Arabic version was translated again into English. The starting and final English versions were compared to confirm that they were similar. Upon validation, a pilot study was conducted on 65 participants who fulfilled the inclusion criteria. The responses obtained from the participants in the pilot study were excluded from the main data analysis. Nevertheless, the suggestions received during the pilot study were incorporated into the final study forms, such as adding items to ask about the primary reason for choosing the pharmacy study program. The questionnaire was then finalized and disseminated for mass data collection.

### Data collection

The final survey was distributed through online media, mainly social media and learning platforms, e.g., Facebook, WhatsApp, and Microsoft Teams messages using the convenience sampling method. The online medium was used to disseminate the survey form to avoid the additional risk of face-to-face interaction during the current COVID-19 restrictions. Periodic reminders were sent during the data collection period.

#### Statistical analysis

Data were analyzed using Statistical Package for the Social Sciences (SPSS-10 Inc., Chicago, IL., USA) version 28.0 (1). The differences between students' demographics, their COVID-19 history and learning experiences as categorical variables, and the total mental wellbeing scores were examined using Mann-Whitney and Kruskal Wallis tests considering they did not meet the assumption of normal distribution (21). A Mann-Whitney U test was run to determine whether mental wellbeing scores differed between all two-group categorical variables. The Kruskal-Wallis test was conducted to determine if there were differences in mental wellbeing scores between all categorical variables of more than two groups. Subsequently, pairwise comparisons were performed using Dunn's (22) procedure with a Bonferroni correction for multiple comparisons. Adjusted *p*-values are presented for these pairwise comparisons. Moreover, a binary logistic regression was used to assess possible associations between the binary outcome variable (i.e., low mental wellbeing and moderate/high mental wellbeing) and the study participants' demographic, lifestyle, medical and academic factors (23, 24). A *p*-value of ≤ 0.05 was set as the significance level for all comparisons.

### Ethical approval

Ethical approval for this study was provided by IIUM (International Islamic University Malaysia) Research Ethics Committee (IREC 2022-081). All participating researchers have obtained administration and/or ethical approvals at their universities, permitting them to conduct the study. The online survey form included the participation information sheet and informed consent. Participants were briefed on the strict confidentiality of their information and the anonymous use of their data for scientific research purposes only. They were told they could withdraw their consent during the study. By approving the consent form, participants were deemed to have consented to participate in this research.

## Results

### Sociodemographic, general health status, and COVID-19-related information

A total of 2,665 responses were received. Participants aged 21.38 ± 1.65, mainly females (68.7%), single (92.5%), and living in urban areas (69.2%). Among the participants, 93.9% reported the absence of any chronic disease. About 18.8% of respondents were overweight, 4.2% were obese, and only 8.9% had a regular exercise routine. Regarding COVID-19-related infection, 36.5% had been infected, approximately 58% had close family members contracted the infection, and 24% had close family members died because of COVID-19. A detailed description of participants' socio-demographic data, general health information, COVID-19 infection history, and vaccination status is provided in Table 1.

**Table 1:** Participants' socio-demographics, lifestyle, and health-related information.

**Characteristic**	***N***	**%**
**Gender**		
Male	835	31.3%
Female	1,830	68.7%
**Marital status**		
Single	2,466	92.5%
Engaged	111	4.2%
Married	88	3.3%
**Area of residence**		
Urban (lives in a city)	1,843	69.2%
Rural (lives in a town or village)	571	21.4%
Urban (lives in a city for education but belongs to rural)	251	9.4%
**Family's household monthly income category**		
Middle-income	2,204	82.7%
Low-income	267	10.0%
High-income	194	7.3%
**Presence of chronic disease or disability**		
No	2,503	93.9%
Yes	162	6.1%
**Exercise status**		
No	973	36.5%
Yes, irregular exercise.	1,454	54.6%
Yes, regular (5 days/week).	238	8.9%
**Smoking status**		
No	2,438	91.5%
Yes	227	8.5%
**Body mass index**		
Normal	1,784	67%
Underweight	267	10.0%
Overweight	501	18.8%
Obese	113	4.2%
**Status of infection with COVID-19**		
No	1,693	63.5%
Yes	972	36.5%
**Status of family member(s) contracted COVID-19 infection**		
No	1,121	42.1%
Yes	1,544	57.9%
**Death of any close family member because of COVID-19 infection**		
No	2,028	76.1%
Yes	637	23.9%
**COVID-19 vaccination status**		
Not vaccinated	219	8.2%
One dose only	180	6.8%
Two doses (full)	1,736	65.1%
Three doses (booster)	530	19.9%

### Academic-related information, challenges, and learning mode

The study respondents were mainly from private universities (59.1%). About 31% were enrolled in Doctor of Pharmacy programs. Enrolment in the Pharmacy program was 56.1% based on their interest and passion, while 29.9% was based on their family recommendation. More than half (58.2%) reported challenges with online learning during the pandemic. Table 2 demonstrates the academic-related information, challenges, and learning mode in the post-pandemic era.

**Table 2:** Participants' academic-related information, challenges, and learning mode.

**Items**	***N***	**%**
**Year of study**		
Year 1	399	15.0%
Year 2	380	14.3%
Year 3	648	24.3%
Year 4	741	27.8%
Year 5	497	18.6%
**The reason behind the choice of a pharmacy study program**		
Interest/passion.	1,495	56.1%
Family/friends recommendation.	797	29.9%
Inspired by a pharmacist I know.	206	7.7%
Only available/reasonable choice.	85	3.2%
Others or no specific reason.	82	3.1%
**Academic performance up to the previous semester/annual exam**		
Excellent	484	18.2%
Very good	943	35.4%
Good	728	27.3%
Moderate	335	12.6%
Fair	175	6.5%
**Undergraduate pharmacy program**		
B.S. Pharm	1,844	69.2%
Pharm D (Doctor of Pharmacy)	821	30.8%
**University type**		
Public	1,091	40.9%
Private	1,574	59.1%
**Facing challenges with online learning during the pandemic**		
No	1,113	41.8%
Yes	1,552	58.2%
**Current learning mode adopted at your faculty**		
Face to face	1,114	41.8%
Hybrid	1,278	48.0%
Online	273	10.2%

### Assessment of mental wellbeing items and categories

**Figure 1:**
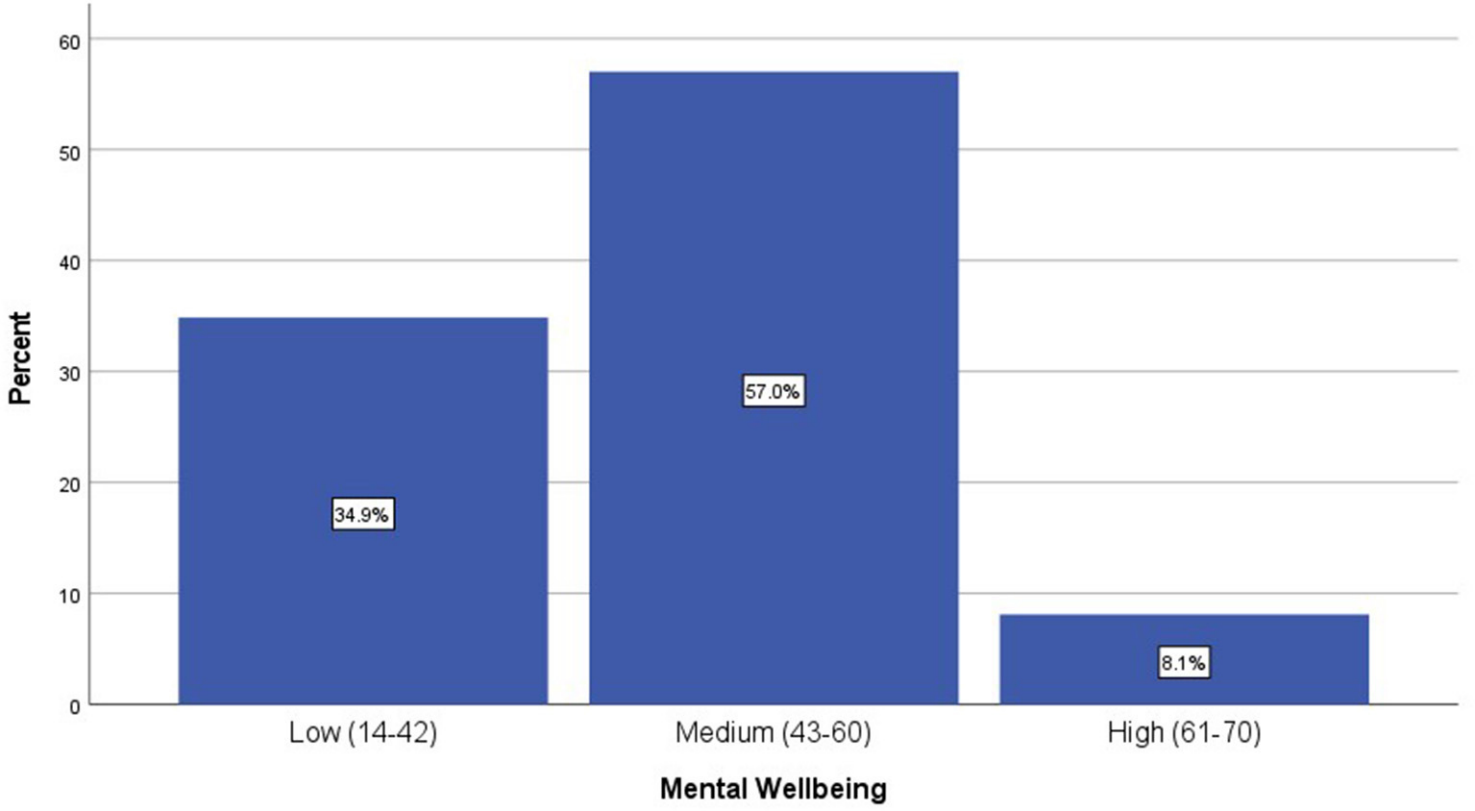
Categories of the mental wellbeing levels among the study participants (*N* = 2665).

The overall mental wellbeing mean (SD) score is 46.5 (10.5). The overall assessment indicated that approximately one-third of participants, 34.9% (*N* = 929), had low mental wellbeing. In contrast, more than half, 57% (*N* = 1,520), of all respondents experienced medium mental wellbeing, and only 8.1% (*N* = 216) of the study participants had high mental wellbeing levels (Figure 1). The individual responses received on the 5-point scale for all 14 items of the mental wellbeing assessment tool are presented in Table 3. The overall look revealed some interesting insights regarding the potential contributing items to the low mental wellbeing levels. For example, item number 3, asking about feeling relaxed, has the highest number of “rarely” and “none of the time” responses (34.5%). This was followed by 27.6% of “rarely” and “none of the time” responses to items related to having the energy to spare and feeling close to other people. Clarity of thinking which is essential for problem-solving in many pharmacy courses, was also negatively affected, as indicated by the figures in the same table. On the other hand, about 24.7% stated they are interested in doing new things all the time.

**Table 3:** Responses to individual items of the mental wellbeing assessment tool (the 14-item WEMWBS).

**Mental wellbeing scale (the 14-item WEMWBS)**	***N*** **(%)**				
	**All of the time**	**Often**	**Some of the time**	**Rarely**	**None of the time**
1. I've been feeling optimistic about the future	441 (16.5)	814 (30.5)	991 (37.2)	292 (11)	127 (4.8)
2. I've been feeling useful	475 (17.8)	789 (29.6)	897 (33.7)	358 (13.4)	146 (5.5)
3. I've been feeling relaxed	174 (6.5)	575 (21.6)	996 (37.4)	635 (23.8)	285 (10.7)
4. I've been feeling interested in other people	384 (14.4)	765 (28.7)	832 (31.2)	437 (16.4)	247 (9.3)
5. I've had the energy to spare	231 (8.7)	673 (25.3)	1,025 (38.5)	496 (18.6)	240 (9.0)
6. I've been dealing with problems well	350 (13.1)	816 (30.6)	988 (37.1)	362 (13.6)	149 (5.6)
7. I've been thinking clearly	318 (11.9)	733 (27.5)	931 (34.9)	491 (18.4)	192 (7.2)
8. I've been feeling good about myself	551 (20.7)	795 (29.8)	806 (30.2)	334 (12.5)	179 (6.7)
9. I've been feeling close to other people	349 (13.1)	742 (27.8)	837 (31.4)	497 (18.6)	240 (9.0)
10. I've been feeling confident	525 (19.7)	785 (29.5)	822 (30.8)	373 (14.0)	160 (6.0)
11. I've been able to make up my own mind about things	604 (22.7)	928 (34.8)	743 (27.9)	268 (10.1)	122 (4.6)
12. I've been feeling loved	499 (18.7)	876 (32.9)	757 (28.4)	342 (12.8)	191 (7.2)
13. I've been interested in new things	657 (24.7)	953 (35.8)	656 (24.6)	262 (9.8)	137 (5.1)
14. I've been feeling cheerful	380 (14.3)	785 (29.5)	908 (34.1)	387 (14.5)	205 (7.7)

Although this study was not planned principally to compare mental wellbeing across countries, it might be interesting to highlight the differences in the percentages of mental wellbeing levels within countries for future studies. Table 4 demonstrates the frequencies and percentages of mental wellbeing levels among participants from every country. It can be observed that the highest percentage of within-country low mental wellbeing levels among pharmacy students was reported for India (43.9%), followed by Libya (43.8%), Malaysia (40.6%), Sudan (37.6%), and Iraq (36.5%). In comparison, the highest percentage of within-country high mental wellbeing levels among pharmacy students was reported for UAE (16.8%), followed by Bahrain (12.8%), Bangladesh (11.5%), Oman (11.4%), and Indonesia (10.5%). These percentages should be reported with caution of lack of generalizability owing to the small number of participants in some of these countries.

**Table 4:** Frequencies and percentages of mental wellbeing levels among participants in the involved countries.

**Country of residence**		**M.W. Categories**			**Total**
		**Low (14–42)**	**Medium (43–60)**	**High (61–70)**	
Bahrain	Count	11	23	5	39
	% Within Country	28.2%	59.0%	12.8%	100.0%
Bangladesh	Count	40	75	15	130
	% Within Country	30.8%	57.7%	11.5%	100.0%
Egypt	Count	200	338	48	586
	% Within Country	34.1%	57.7%	8.2%	100.0%
India	Count	112	123	20	255
	% Within Country	43.9%	48.2%	7.8%	100.0%
Indonesia	Count	73	114	22	209
	% Within Country	34.9%	54.5%	10.5%	100.0%
Iraq	Count	65	104	9	178
	% Within Country	36.5%	58.4%	5.1%	100.0%
Jordan	Count	64	143	17	224
	% Within Country	28.6%	63.8%	7.6%	100.0%
Libya	Count	14	16	2	32
	% Within Country	43.8%	50.0%	6.3%	100.0%
Malaysia	Count	80	111	6	197
	% Within Country	40.6%	56.3%	3.0%	100.0%
Oman	Count	17	45	8	70
	% Within Country	24.3%	64.3%	11.4%	100.0%
Pakistan	Count	94	168	16	278
	% Within Country	33.8%	60.4%	5.8%	100.0%
Saudi Arabia	Count	64	107	19	190
	% Within Country	33.7%	56.3%	10.0%	100.0%
Sudan	Count	64	95	11	170
	% Within Country	37.6%	55.9%	6.5%	100.0%
United Arab Emirates	Count	31	58	18	107
	% Within Country	29.0%	54.2%	16.8%	100.0%
Total	Overall Count	929	1,520	216	2,665
	% Within All Countries	34.9%	57.0%	8.1%	100.0%

### Factors associated with higher mental wellbeing scores

Higher mental wellbeing levels were reported among males (*p* < 0.001), those in private universities compared to those in public universities (*p* = 0.012), and among those who did not face challenges with online learning during the pandemic compared to those who did face challenges (*p* < 0.001). Higher mental wellbeing levels were reported for irregular and regular exercisers compared to no exercise (*p* < 0.001). Also, for those with normal body mass index (BMI) compared to overweight (*p* = 0.014) and underweight (*p* = 0.001). Additionally, among those who chose the pharmacy program based on their interest and passion compared to family recommendations (*p* < 0.001) and those who chose the pharmacy program as their only available/reasonable choice (*p* = 0.013). Finally, higher mental wellbeing scores were reported among students with higher grades, such as excellent and very good, compared to those with lower grades, such as moderate and fair. Table 5 presents all associated factors that correlate with higher mental wellbeing scores.

**Table 5:** Factors associated with significant differences in the mental wellbeing scores.

**Factor**	**Category with higher mental wellbeing scores (mean rank)**	**Category with lower mental wellbeing scores (mean rank)**	***p*-value**
Gender	Males (1,441)	Females (1,283)	*p* < 0.001^*^
Presence of disease	Those without chronic disease (1,349)	Those with chronic disease (1,084)	*p* < 0.001^*^
University type	Students in private universities (1,364)	Students in public universities (1,287)	*p* = 0.012^*^
Facing challenges with online learning	Those who did not face challenges (1,405)	Those who did face challenges (1,281)	*p* < 0.001^*^
Exercise	M.W. scores were statistically significantly different between pairwise compared physical activity groups.		*p < 0.0*01^#^
	Regular exercise (1,496)	No exercise (1,203)	*p* < 0.001
	Irregular exercise (1,393)	No exercise (1,203)	*p* < 0.001
BMI	MW scores were statistically significantly different between pairwise compared BMI groups.		*p* < 0.001^#^
	Normal (1,378)	Underweight (1,189)	*p* = 0.001
	Normal (1,378)	Overweight (1,250)	*p* = 0.014
Reason for choosing the pharmacy program.	M.W. scores were statistically significantly different between pairwise compared reasons for choosing the pharmacy program.		*p* < 0.001^#^
	Interest/passion (1,430)	Family/friends recommendation (1,175)	*p* < 0.001
	Interest/passion (1,430)	Only available/reasonable choice (1,154)	*p* = 0.013
	Inspired by a pharmacist I know (1,362)	Family/friends recommendation (1,175)	*p* = 0.018
Academic performance	M.W. scores were statistically significantly different across pairwise compared academic performance grades.		*p* < 0.001^#^
	Excellent (1,399)	Moderate (1,185)	*p* = 0.001
	Excellent (1,399)	Fair (1,157)	*p* = 0.004
	Very good (1,360)	Moderate (1,185)	*p* = 0.003
	Very good (1,360)	Fair (1,157)	*p* = 0.013
	Good (1,362)	Moderate (1,185)	*p* = 0.005
	Good (1,362)	Fair (1,157)	*p* = 0.016

* Mann-Whitney test (significance measure at *P* < 0.05).

# Kruskal-Wallis test (adjusted *p*-values are presented for the pairwise comparisons).

Table 6 shows the binary logistic regression results concerning the odds of having higher mental wellbeing levels. It was observed that males were more likely to have higher mental wellbeing than females (AOR: 1.34; CI 95%: 1.11–1.61; *p* < 0.01). Study participants with no chronic illnesses were more likely to have higher mental wellbeing than those with chronic diseases (AOR: 2.01; CI 95%: 1.45-2.80; *p* < 0.001). It was also seen that those participants who did not engage in any exercise were less likely to have higher mental wellbeing when compared to those who were involved in exercise (AOR: 0.71; CI 95%: 0.52–0.98; *p* = 0.04). The study sample who had interest/passion for pharmacy (AOR: 1.69; CI 95%: 1.07–2.68; *p* = 0.02), and those who known pharmacists inspired (AOR: 1.81; CI 95%: 1.06–3.12; *p* = 0.03), were more likely to have higher mental wellbeing in comparison with those who had no specific reason for their choice to study pharmacy. The participants with good (AOR: 1.49; CI 95%: 1.05–2.11; *p* = 0.02), very good (AOR: 1.57; CI 95%: 1.12–2.22; *p* = 0.01), (or) excellent academic performance (AOR: 1.87; CI 95%: 1.29–2.70; *p* = 0.001) were more likely to have higher mental wellbeing as compared to those with fair academic performance. Finally, the study participants who studied in public universities were less likely to have higher mental wellbeing when compared with those in private universities (AOR: 0.82; CI 95%: 0.69–0.97; *p* = 0.02).

**Table 6:** Predictors of higher mental wellbeing among pharmacy students as identified by the binary logistic regression.

	**AOR**	**95% C.I**.		*p*-value^*^
		**Lower**	**Upper**	
**Gender**				
Male	1.34	1.11	1.61	0.002
Female				Ref
**Presence of chronic diseases**				
No	2.01	1.45	2.80	*p* < 0.001
Yes				Ref
**Exercise status**				*p* < 0.001
No	0.71	0.52	0.98	0.04
Irregular exercise	1.10	0.81	1.50	0.56
Regular exercise				Ref
**Body mass index (BMI)**				0.001
Normal	1.17	0.78	1.76	0.45
Underweight	0.76	0.48	1.21	0.25
Over-weight	0.85	0.55	1.31	0.46
Obese				Ref
**Reason for choosing the pharmacy program**				*p* < 0.001
Interest/passion	1.69	1.07	2.68	0.02
Recommendation by family/friends	1.01	0.63	1.62	0.97
Inspired by known pharmacists	1.81	1.06	3.12	0.03
Only available choice	0.93	0.50	1.74	0.82
Others/no specific choice				Ref
**Academic performance**				0.002
Excellent	1.87	1.29	2.70	0.001
Very good	1.57	1.12	2.22	0.01
Good	1.49	1.05	2.11	0.02
Moderate	1.15	0.78	1.69	0.47
Fair				Ref
**Type of university**				
Public	0.82	0.69	0.97	0.02
Private				Ref
**Online challenges**				
No	1.15	0.97	1.36	0.11
Yes				Ref

* Significant (*P*-value < 0.05).

## Discussion

The present study revealed a thorough assessment of mental wellbeing levels and identified a group of associated factors that are thought to correlate with students' mental wellbeing. With this, our study could be the most recent work that reports on the mental wellbeing of pharmacy students across various Asian and Middle Eastern countries with a relatively large sample (*N* = 2,665). The assessment of mental wellbeing has shown that approximately one-third of the study population had low mental wellbeing levels. More interestingly, the findings revealed significant differences in the mental wellbeing scores based on several demographic, lifestyle, and academic-related factors.

One in three students had a low level of mental wellbeing, which was our most alarming finding. Looking at the cut-off points, low mental wellbeing is between 14 and 42. Therefore, students who answered positively to some items, not all, or answered some of the time to all items will be classified under this category. Also, 57% were in the medium category (43–60), where they could have answered many questions positively in a pattern more frequent than in the first category. The presented overall mental wellbeing scores, categories (Figure 1), and detailed responses (Table 3) complement each other to assess students' mental wellbeing comprehensively. Mental wellbeing refers to the capacity of an individual to maintain a state of feeling good and functioning well, which encompasses more than the treatment or prevention of mental illness (2). A previous small Australian study that looked at predictors of mental wellbeing assessed using the WEMWBS reported medium wellbeing for most participants as reported in the present study (6). In a relatively smaller U.S. study that looked at the mental health among PharmD students, more than 25% of the participants were at high risk of mental health issues such as depression and general anxiety provoked mainly by academic-related stress (25). Another study in the U.S. also reported that 50% of PharmD students had general anxiety triggered by academic and family distress (26). In a study conducted in Egypt among 164 students, the prevalence of anxiety and depression were 29 and 51%, respectively. A study of 750 pharmacy and medical students in Iraq found that 45.9% had scores that indicated depression symptoms, and 52.1% of the participants had scores that indicated anxiety symptoms (27). We acknowledge that the mental wellbeing assessment is not generally an assessment for a particular mental health issue; instead, it might help to identify those with low mental wellbeing who are more prone to mental health issues (2). The reported percentage of low mental wellbeing (35%) in the present study can be considered average compared to the previously reported values (25–50%) owing to the relatively larger sample size in our study compared to other single-centered studies and the difference in the assessment tools across various studies. However, the fact that one-third of our sizable population (*N* = 2,665) experienced low mental wellbeing should be a trigger for concern.

Given the concerningly low level of mental wellbeing, pharmacy schools may need to re-evaluate the support and counseling services that should contribute to preserving the wellbeing of their students. From students' perspectives, universities play an essential role in enhancing their mental wellbeing through academic practices such as academic instructions, teaching practices, and course design, in addition to the support, culture, environment, and communication (28). Pharmacy schools might need to target students with lower mental wellbeing through initiatives to improve mental health literacy, promote resilience, and encourage students to seek help while facing difficulties in coping with their academic life demands (29). An excellent example of a framework to promote students' wellbeing within the university environment was proposed in Australia and identified five main strategic initiatives as increasing community involvement and awareness, creating interactive curricula and learning experiences, educating students about mental health and how to take responsibility for their wellbeing; and providing them with easy access to high-quality care (30). The capacity of academic institutions to apply these strategic initiatives could be different across public and private universities, affected mainly by the typically increased number of students in public universities. For example, the undergraduate pharmacy student population in one public school in one of the participating countries exceeds one thousand students, making the provision of individual support initiatives challenging (31). This might explain the lower mental wellbeing levels among public university students compared to their private university counterparts identified in our study. This could be seen in the context of the challenges of maintaining the appropriate intensity and quality of the students' support services at public pharmacy schools (26). Recognizing that most academic institutions have returned to their norm before the pandemic, it might be the right time to restructure and customize strategic plans that consider aspects of the framework to improve students' mental health and wellbeing (30).

Concerning the factors associated with mental health issues, previous reports revealed an association between mental health issues with academic distress (25) and family distress (26). Although our study did not assess family distress, our findings provide a more comprehensive list of several demographic, health-related, and academic-related factors that seem to have a role in shaping the students' mental wellbeing status. For example, the present study highlighted the gender-based difference in the mental wellbeing status where females were more prone to have a low level of mental wellbeing. Coincidently, a study from Saudi Arabia highlighted those female students were more likely to experience psychological distress (32). Similarily, a large-scale U.S. study among medical students also highlighted females as more prone to have poor mental wellbeing (33). In an Irish study aimed to explain the relatively consistent pattern of poor mental health among females compared to males, it has been highlighted that this pattern could be explained partially by differences in employment, marital status, and club memberships. However, part of this difference pattern is still yet to be fully elucidated (34). As our study involved only students and few of them are married, it might trigger planning for further analysis among the students to explore factors contributing to this difference in mental wellbeing levels. As a practical recommendation, the gender differences in mental wellbeing could be a relevant point to consider in planning mental health support services where more focus and efforts could be directed to female students, usually a majority in pharmacy schools globally. An example of these targeted interventions for female students to enhance their mental wellbeing is a six-week aerobic training program among Iranian female students that was associated with a significant impact on their overall mental health (35).

Interestingly, our findings underpinned the positive role of physical activity and maintaining normal weight on overall mental wellbeing. Compared to no exercise, those engaged in exercise, even if irregularly, were more likely to have better mental wellbeing. There is a well-established relationship between physical activity and mental health, where engagement in aerobic exercise could reduce symptoms of depression significantly, whereas excessive physical activity could generate psychological symptoms (36). Moreover, the present study showed that students with chronic diseases were less likely to have higher mental wellbeing. A nationwide French survey highlighted that participants with disability were more likely to experience a negative impact on mental wellbeing amid the pandemic (37).

Additionally, an Austrian study evaluating risk factors for psychological distress showed that the participants' BMI played no significant role (38). On the other hand, the current study findings suggest that maintaining a normal BMI contributes to students' mental wellbeing. Students who maintained normal BMI had relatively higher mental wellbeing levels than participants with under-weight and over-weight. This may highlight the significance of encouraging students to engage in moderate exercise, sign up for sports competitions and university sports teams and provide them with other resources to help them maintain a healthy lifestyle, both of which the university setting can help facilitate (28).

Furthermore, from an academic perspective, students' mental wellbeing was linked to facing challenges with online learning, academic performance, and initial interest in the pharmacy program. Although an earlier study in Kazakhstan highlighted improved mental health of medical students while transitioning from traditional to online learning (39), our findings revealed that online learning challenges were linked to lower mental wellbeing. Furthermore, students with lower academic performance grades tended to be more prone to having poor mental health (39). This is consistent with our results that students with higher academic performance had higher mental wellbeing levels.

Finally, our findings showed that students who had an initial interest in the pharmacy program were more likely to have better mental wellbeing than their counterparts who had entered the program mainly because of family recommendations or as the only feasible option. This might shed light on the intriguing idea that the wellbeing of pharmacy students could have started before they enter the program through professional identity formation and outreach programs to cultivate the interest in becoming students professionally (40).

As a central point, our relatively large study confirms that students' mental wellbeing is multifactorial and that achieving optimal outcomes typically requires concurrently addressing multiple factors. However, additional research is needed to examine the design and effectiveness of interventions most likely to improve students' mental wellbeing and assist them in achieving their personal and academic goals.

## Limitations

The main limitation of the present study is the inconsistent samples across different countries attributed to various factors. These variations did not allow proper cross-country comparisons and hindered the provision of country-specific recommendations. Furthermore, the cross-sectional design can only provide a snapshot of the actual scenario in these settings. Moreover, the convenience sampling method is a non-probability sampling technique, which may limit the generalizability of the findings to other students in the participating countries. Finally, the self-reported nature of the online surveys may introduce bias, such as the information related to the monthly income “self-reported according to each country's specific classification for low, middle, and high-income” and the body mass index “self-reported according to ranges provided in the survey”.

## Implications

This could be one of the few studies involving a large and diverse pharmacy student population from 14 countries. This study identified a comprehensive list of factors significantly related to mental wellbeing. The findings reaffirmed the multifaceted nature of pharmacy students' mental wellbeing and paved the way for further coordinated and multifaceted interventions to be implemented by pharmacy schools to improve their students' mental wellbeing. Academic institutions should set programs to enhance their students' mental wellbeing by revising academic practices, learning environments and support services. More specifically, efforts should be directed into programs to improve mental health literacy, promote resilience, and facilitate ways for students to seek help while facing challenges in coping with their academic life stressors.

## Conclusion

More than a third of the participants had low mental wellbeing levels. Several demographic, lifestyle, medical, and academic factors were associated with mental wellbeing levels. Careful consideration of these factors and their integration into the pharmacy schools' plans for student support services and academic advising would be essential to improve students' mental wellbeing.

## Data availability statement

The original contributions presented in the study are included in the article/supplementary material, further inquiries can be directed to the corresponding author.

## Ethics statement

The studies involving human participants were reviewed and approved by IIUM Research Ethics Committee (IREC 2022-081). The Ethics Committee waived the requirement of written informed consent for participation.

## Author contributions

All authors listed have made a substantial, direct, and intellectual contribution to the work and approved it for publication.

## Conflict of interest

The authors declare that the research was conducted in the absence of any commercial or financial relationships that could be construed as a potential conflict of interest.

## Publisher's note

All claims expressed in this article are solely those of the authors and do not necessarily represent those of their affiliated organizations, or those of the publisher, the editors and the reviewers. Any product that may be evaluated in this article, or claim that may be made by its manufacturer, is not guaranteed or endorsed by the publisher.
